# Novel Therapeutic Targets in Acute Myeloid Leukemia (AML)

**DOI:** 10.1007/s11912-024-01503-y

**Published:** 2024-03-19

**Authors:** Michael Wysota, Marina Konopleva, Shane Mitchell

**Affiliations:** 1 Department of Oncology, Montefiore Medical Center, 111 East 210 Street, Bronx, NY 10467 USA; 2 Montefiore Medical Center/Albert Einstein College of Medicine, Albert Einstein College of Medicine, Jack and Pearl Resnick Campus, Ullmann Building, 1300 Morris Park AvenueRoom 915, Bronx, NY 10461 USA; 3 Albert Einstein College of Medicine, Bronx, NY USA

**Keywords:** Acute myeloid leukemia, Novel therapeutics, Molecular targets, AML, Immune therapy

## Abstract

**Purpose of Review:**

This review seeks to identify and describe novel genetic and protein targets and their associated therapeutics currently being used or studied in the treatment of acute myeloid leukemia (AML).

**Recent Findings:**

Over the course of the last 5–6 years, several targeted therapies have been approved by the FDA, for the treatment of both newly diagnosed as well as relapsed/refractory AML. These novel therapeutics, as well as several others currently under investigation, have demonstrated activity in AML and have improved outcomes for many patients.

**Summary:**

Patient outcomes in AML have slowly improved over time, though for many patients, particularly elderly patients or those with relapsed/refractory disease, mortality remains very high. With the identification of several molecular/genetic drivers and protein targets and development of therapeutics which leverage those mechanisms to target leukemic cells, outcomes for patients with AML have improved and continue to improve significantly.

## Introduction

AML is the most common acute leukemia in adults, with an estimated 20,380 new cases and 11,310 deaths in the USA in 2023 alone [1]. The standard of care for the treatment of AML for the last 50 years has been high-intensity induction chemotherapy followed by additional consolidation chemotherapy and, depending on the patient’s risk category and overall fitness, allogeneic stem cell transplant. After induction chemotherapy, complete remission (CR) is seen in approximately 73%, 66%, and 45% of patients in ELN-2022 favorable, intermediate, and adverse risk groups respectively [2]. Five-year progression free survival (PFS) was estimated at 52%, 32%, and 16%, and 5-year overall survival (OS) was 55%, 34%, and 15% respectively in the same population. This is similar to other studies showing 5-year OS rates of 62% in core binding factor (CBF) AML and 21% in all other AML patients [3]. Improvements have been made to the induction chemotherapy regimen in both younger and older patients, which has resulted in higher CR rates and better relapse-free and overall survival [4]. Despite this, there is still significant room for improvement toward achieving better long-term outcomes. For patients older than 60 and patients with relapsed/refractory disease, survival is particularly poor with 5-year OS 4–18% and ~ 10% respectively [5, 6].

**Fig. 1:**
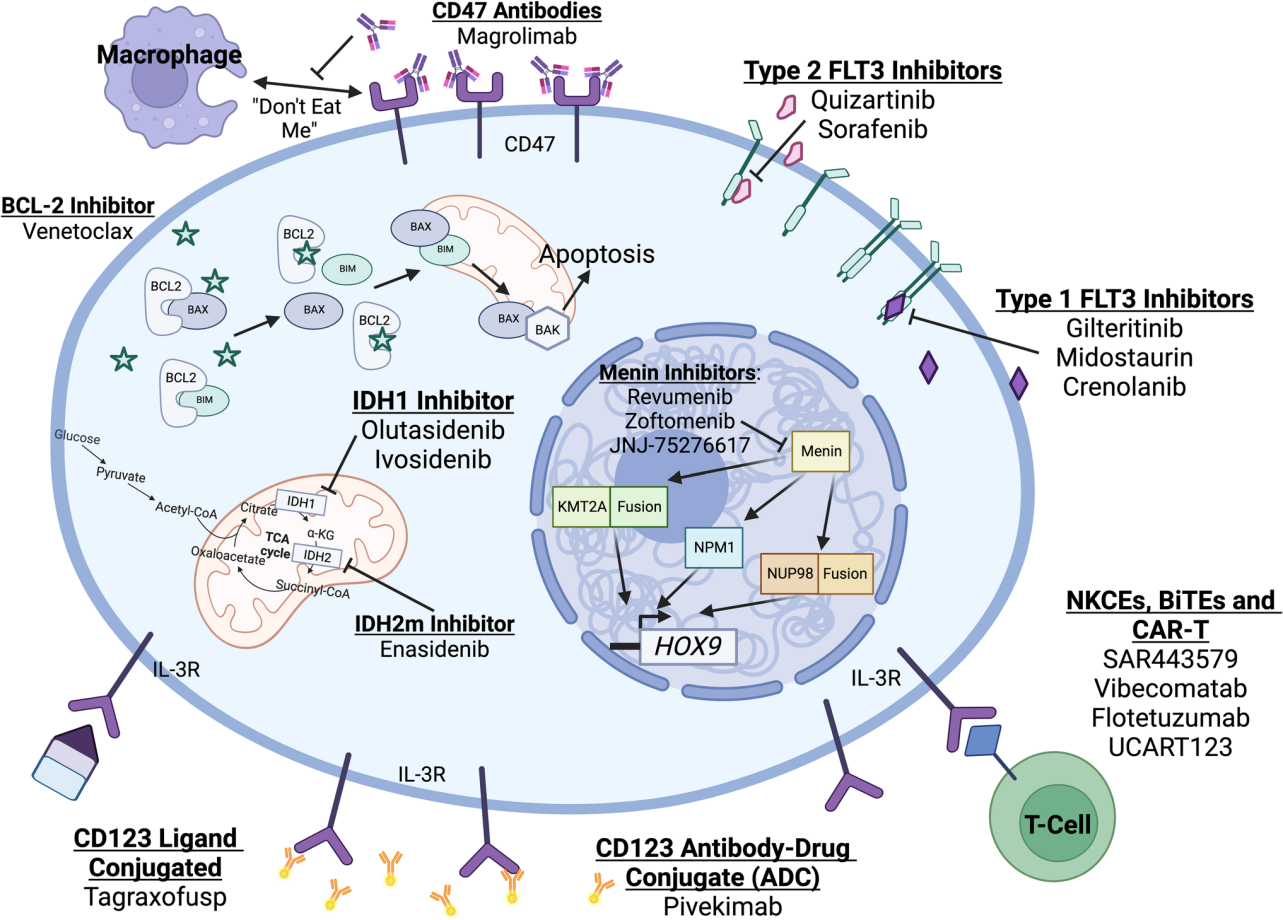
Novel therapeutics and their molecular targets which have been studied for treatment of acute myeloid leukemia

Within the last 5–6 years, however, the FDA has approved several targeted therapies which have demonstrated efficacy in treating AML in both the front line and relapsed/refractory settings (Tables 1 and 2). In addition to these approved therapies, there are several additional targets currently under investigation in AML, which have also shown promise (Fig. 1). This article is dedicated to discussing several molecular mechanisms contributing to AML pathogenesis and to review current research into how these mechanisms are being targeted in the treatment of AML.

**Table 1:** Novel therapeutic targets in AML

Target	Gene mutated	Mechanism	Prognostic implication
BCL2	None	Anti-apoptotic	None
FLT3	FLT-3 ITD FLT-3 TKD	Constitutive activation of receptor tyrosine kinase	ELN 2022 intermediate risk for ITD None for TKD
IDH1	IDH1	Altered Krebs cycle and increased 2-HG production, resulting in altered gene expression profile	Variable
IDH2	IDH2	Altered Krebs cycle and increased 2-HG production, resulting in altered gene expression profile	Variable
Menin	KMT2A fusion NPM1 NUP98 fusion	Altered expression of Hox genes important for leukemogenesis	ELN 2022 Intermediate for 9;11 fusion Poor risk for other fusions ELN 2022 Good risk for NPM1 without other mutations No ELN prognostic risk for NUP98 fusions though thought to be adverse risk factor
CD123	None	Cell surface marker overexpressed on AML blast cells and leukemia stem cells	None
CD47	None	“Don’t-eat-me signal” for macrophages	None

**Table 2:** Novel therapeutics in AML

Target	Therapeutics	FDA-approved therapies
BCL2	Venetoclax	*Venetoclax* In combination with azacitidine, decitabine, or low-dose cytarabine (LDAC) for newly-diagnosed acute myeloid leukemia (AML) in adults 75 years or older, or who have comorbidities precluding intensive induction chemotherapy [10]
FLT3	Midostaurin Gilteritinib Quizartinib Sorafenib Crenolanib	*Midostaurin* In combination of 7 + 3 chemotherapy in adult patients with FLT3m AML [21] *Gilteritinib* As monotherapy for treatment of R/R FLT3m AML [22] *Quizartinib* In combination of 7 + 3 chemotherapy in ND adult patients with FLT3m AML [26••]
IDH1	Ivosidenib Olutasidenib	*Ivosidenib* As monotherapy for adult patients with relapsed or refractory acute myeloid leukemia (AML) with a susceptible IDH1 mutations [48] In combination with azacitidine for newly diagnosed acute myeloid leukemia (AML) with a susceptible IDH1 mutation [49] *Olutasidenib* As monotherapy adult patients with relapsed or refractory acute myeloid leukemia with a susceptible IDH1 mutation [51]
IDH2	Enasidenib	*Enasidenib* As monotherapy for adult patients with relapsed or refractory acute myeloid leukemia with an IDH2 mutation [45]
Menin	Revumenib Ziftomenib JNJ-72576617	Pending
CD123	**ADC** Pivekimab **BITE** Vibecomatab Flotetuzumab **CAR-T** UCART123V1.2 **Biologic** Tagraxofusp	None
CD47	Magrolimab	None

## BCL-2

The B cell lymphoma 2 (BCL-2) family of proteins comprises 12 members with either pro- or anti-apoptotic functions. The change in expression of these proteins either promotes survival or commits a cell to apoptosis. BCL-2 is one member of this protein family. It is a cytoplasmic protein which regulates apoptosis through control of mitochondrial membrane permeabilization (MOMP). BCL-2 and related proteins prevent MOMP by sequestering pro-apoptotic proteins, which results in decreased permeability of the mitochondrial membrane, resulting in the prevention of the release of cytochrome C from mitochondria and activation of apoptosome [7]. BCL-2 has been shown to important to the survival and proliferation of leukemic cells initially in a murine model [8]. The small molecule BCL-2 inhibitor Venetoclax, initially explored mostly in lymphoid malignancies particularly CLL, has demonstrated significant activity in AML in multiple clinical trials.

Venetoclax was initially studied as monotherapy in patients with R/R AML, with some single agent activity demonstrated. However, responses were short lived with median PFS of 2.5 months. Resistance to Venetoclax was shown in this study, in part due to leukemic blasts’ dependence on other antiapoptotic members of the BCL-2 family (BCL-XL and MCL-1) [9].

Given limited single agent activity and the potential of this agent to “prime” AML blasts to chemotherapy and targeted therapies, Venetoclax has also been studied and is currently in trials in combination with several other agents. Most famously, Venetoclax was studied in combination with the hypomethylating agent (HMA) Azacitidine for AML patients unfit to receive intensive induction chemotherapy, in the VIALE-A study [10]. Patients received daily Venetoclax, titrated to a 400 mg dose over 3 days, along with standard dose Azacitidine (75 mg/m2 for 7 days) vs Azacitidine alone. Overall survival was prolonged with the combination (14.7 months vs 9.6 months). Patients also had higher rates of complete remission/complete remission with incomplete count recovery (CR/CRi) (64.7% vs 22.8%) and measurable residual disease (MRD) negativity (23.4% vs 7.6%). The most common Grade 3, or higher, toxicities were neutropenia and febrile neutropenia, and tumor lysis syndrome (TLS) was rare (1%). Notably, the drug combination did not appear to significantly affect survival outcomes in patients with TP53 mutations. This ultimately resulted in the FDA approval of Venetoclax in combination with Azacitidine for treatment of newly diagnosed (ND) AML in patients unfit for intensive induction.

Venetoclax was also studied in combination with low dose cytarabine (LDAC) in the VIALE-C trial. 211 patients were randomized to LDAC at 20 mg/m2 daily for 10 days + / − Venetoclax (titrated up to 600 mg). OS was not statistically significantly prolonged though trended toward significance (7.2 vs 4.1 months) and CR/CRi rates were improved (48% vs 13%) [11].

There are now additional efforts to combine Venetoclax with intensive chemotherapy in several early phase trials. Venetoclax has been combined with Fludarabine, Cytarabine, GCSF, Idarubicin (FLAG-IDA), Cladribine, Idarubicin, Cytarabine (CLIA), Standard Daunorubicin and cytarabine (7 + 3) along with several other regimens, in both ND and R/R AML. CR and MRD negativity rates have been very encouraging (89–94% and 82–93% respectively) [12••, 13]. Treatment-related side effects profiles have also been similar to those previously reported with intensive induction regimens without Venetoclax. While comparative trials are lacking, there are several phase 3 trials of Venetoclax + intensive induction chemotherapy currently enrolling (NCT04628026).

Finally, Venetoclax has also been studied in combination with many of the other targeted agents presented here and will be discussed in the respective sections.

## FLT-3

FMS-related tyrosine kinase 3 (FLT-3) is a member of the class III receptor tyrosine kinase family, which contains several other members including PDGFR, and c-KIT. FLT-3 is expressed selectively on CD34 + hematopoietic stem cells (HSCs) and is responsible for regulating the early stages of hematopoiesis. FLT-3 binds to its associated ligand at the cell membrane resulting in FLT-3 dimerization and activation of the cytoplasmic tyrosine kinase domain (TKD), which leads to downstream signaling through several pathways including PIK3A, RAS, MAPK/ERK [14]. FLT-3 mutations are common in AML and can be present in approximately 30% of cases. There are two described classes of FLT-3 mutations (FLT-3 m), internal tandem duplications (ITD), which are in-frame duplications of 3–400 base pairs, and TKD point mutations, both of which result in constitutive activation of the receptor and uncontrolled proliferation of leukemic stem cells (LSCs) [15]. FLT-3 mutations have previously been associated with higher white blood cell (WBC) count at diagnosis, higher peripheral blood (PB) and bone marrow (BM) blast counts, and shorter PFS and OS compared with patients not harboring these mutations [16–18]. Notably FLT-3 ITD mutations have clear implications for disease biology and prognosis, however the role of TKD mutations remains controversial [7, 19].

There have been several FLT-3 targeting agents developed to inhibit constitutively active FLT-3 in leukemic stem cells in patients with FLT-3 m AML. The first generation of drugs Midostaurin and Sorafenib are multikinase inhibitors which inhibit FLT-3 in addition to several other off-target kinases. Second generation FLT-3 inhibitors Gilteritinib, Quizartinib, and Crenolanib have greater specificity and potency than first generation inhibitors. Inhibitors are divided into two types. Type 1 inhibitors (Gilteritinib, Midostaurin, Crenolanib) inhibit the active conformation of FLT-3 while Type 2 inhibitors (Quizartinib, Sorafenib) inhibit the inactive conformation [20].

Since 2017, the standard of care for fit patients with ND FLT-3 m AML has been 7 + 3 chemotherapy with the addition of Midostaurin, based on the results of the Phase III RATIFY trial, which showed prolonged survival when Midostaurin was added to intensive chemotherapy [21]. Since that time, many additional trials have been done evaluating FLT3 inhibitors in AML.

Gilteritinib has been approved as monotherapy for treatment of R/R AML based on the results of the 2019 phase 3, open-label, randomized control ADMIRAL trial [22]. This trial randomized patients with R/R FLT-3 ITD and TKD mutated AML to either continuous Gilteritinib 120 mg daily or physician’s choice of one of several salvage regimens. OS was higher in the Gilteritinib arm (9.3 vs 5.6 months). CR/CRi rate was also higher (34% vs 15%). Common adverse events with Gilteritinib include cytopenias and serum aminotransferase elevations. Gilteritinib even has activity in the R/R setting in patients previously treated with 7 + 3 + Midostaurin (CR rate of 58%) [23]. Several studies of Gilteritinib in the front-line setting have also been performed. Gilteritinib was combined with standard 7 + 3 induction followed by HiDAC maintenance and 2 years of Gilteritinib maintenance in a phase 1 trial with promising results. Composite CR (CRc = CR + CRi + Complete Remission with incomplete platelet recovery CRp) was 89% for patients with FLT-3 m AML with median OS of 45 months [24]. The LACEWING study evaluating Gilteritinib + Azacitidine in FLT-3 m AML patients unfit for intensive induction therapy resulted in higher CRc rates but no significant OS benefit when compared to Azacitidine alone [25].

In July 2023, the FDA-approved Quizartinib for use in ND AML in combination with 7 + 3 induction chemotherapy. This approval was based on the randomized phase 3, double-blind, placebo-controlled QuANTUM-First trial [26••]. This trial enrolled 539 patients with FLT-3 ITD mutated AML and randomized them to 7 + 3 induction followed by high-dose cytarabine consolidation + / − allo-transplant alone or with Quizartinib 40 mg on days 8–21 of induction and daily with consolidation followed by maintenance Quizartinib for up to 3 years. The addition of Quizartinib resulted in significantly longer OS (31.9 vs 15.2 months). Patients had similar rates of CR/CRi but, in those patients achieving CR, MRD negativity rates were higher. Notably, Quizartinib increased rates of QT prolongation, torsades de pointes, and cardiac arrest and therefore is available only through a Risk Evaluation and Mitigation Strategy (REMS). Quizartinib has also been evaluated as monotherapy in the relapsed refractory setting. In the Phase 3 randomized, controlled, open-label Quantum-R study Quizartinib, at 60 mg daily, was evaluated against one of several salvage chemotherapy regimens in patients with FLT-3 m R/R AML. OS was longer in the Quizartinib group (6.2 vs 4.7 months). CR/CRi rates were also higher in the Quizartinib group (48% vs 27%) though responses were short lived [27]. Interestingly, significantly more patients in the Quizartinib group went on to receive allo-transplant (32% vs 11%) and, in a post hoc analysis, patients who underwent allo-transplant had significantly longer OS than those that did not 12.2 vs 4.4 months.

Sorafenib has yet to be FDA approved for treatment of AML, though it has been studied in ND AML, with or without FLT-3 mutations, in combination with intensive chemotherapy in the phase 2 double blind, placebo-controlled SORAML trial. In this trial, patients were randomized to 7 + 3 induction chemotherapy followed by 3 cycles of high dose cytarabine consolidation + / − Sorafenib followed by Sorafenib maintenance for 12 months. Addition of Sorafenib resulted in improvement in 5-year event free survival (EFS) (41% vs 27%) and relapse free survival (RFS) (53% and 36%) but did not significantly prolong OS possibly driven by more aggressive/resistant relapses in patients who initially received Sorafenib. Notably, only 17% of patients in this study had FLT-3 ITD mutations [28]. Sorafenib was more recently evaluated in the front-line setting in patients with FLT-3 ITD mutations. In the prospective Phase 2 double-blind placebo controlled ALLG AMLM16 study, the addition of Sorafenib to 7 + 3 induction did not improve EFS and OS in patients with ND FLT-3 m AML [29]. Contradictorily, a retrospective study 183 patients with FLT-3 ITD were treated with various induction chemotherapies with or without Sorafenib. In both the unmatched and matched cohorts, the addition of Sorafenib resulted in higher EFS and OS, indicating benefit of Sorafenib in the front-line setting [30].

Several studies of FLT-3 inhibitors in combination with standard of care HMA + Venetoclax have been performed for elderly patients with FLT-3 m AML unfit for intensive chemotherapy. The combination of Quizartinib, Decitabine, and Venetoclax was studied in Phase I/II trial in a small group of patients with ND, unfit for intensive therapy, and R/R FLT-3 m AML with excellent CR/CRi rates (100% in ND and 78% in R/R) [31]. OS in R/R patients was 7.6 months. Gilteritinib was also studied in combination with Azacitidine/Venenetoclax, in a Phase I/II trial, in a similar patient population. In the ND population, the CR rate was 95% and 81% achieved MRD negativity. One year OS in the same group was 80%. In the R/R cohort, the CR/CRi rate was 37% with 43% achieving MRD negativity [32•]. The phase 1/2 VICEROY study of this triple combination is ongoing, with the goal of further optimizing this regimen in patients with ND FLT-3 m AML (NCT05520567).

The current standard of care for most fit patients with FLT-3 m AML after induction chemotherapy, in complete remission (CR1), is to offer allogeneic stem cell transplant (Allo-SCT). Despite this aggressive treatment strategy, relapse rates for post-transplant patients with FLT-3 m AML remain high relative to those patients with wild type FLT-3 [33]. Several trials evaluating FLT-3 inhibitors as maintenance after allo-SCT have also been performed. The SORAMIN and RADIUS trials evaluated Sorafenib and Midostaurin respectively. In the Phase II, placebo-controlled SORAMIN trial 83 adult patients with FLT3-ITD mutated AML post allo-stem cell transplant, were randomized to maintenance Sorafenib vs placebo [34]. Sorafenib was dose escalated up to 400 mg twice daily for up to 2 years. Patients in the Sorafenib arm had longer RFS (NR vs 30.9 m), and the hazard ratio for relapse or death was 0.39 (95% CI, 0.18 to 0.85). Patients who had had no MRD pretransplant and those who had detectable MRD post-transplant derived the most benefit. Notably, only 9 of the patients in the trial received Midostaurin prior to transplant so it may be difficult to extrapolate this data for patients treated with current standard of care induction chemotherapy + FLT-3 inhibitors. The RADIUS trial, published soon after SORAMIN, evaluated post-transplant maintenance with Midostaurin and found a numerical, but not statistically significant, 18-month RFS difference (89% vs 76%) favoring Midostaurin over placebo [35]. Finally, the results of the MORPHO trial evaluating post-transplant Gilteritinib were recently presented [36••]. 356 patients were randomized to Gilteritinib vs placebo. In the intention to treat population Gilteritinib did not significantly prolong 2-year RFS, though in pre-specified subgroup analysis, patients with detectable MRD, who were treated with Gilteritinib, had significantly decreased risk of relapse (HR 0.51, *p* = 0.0065) compared to placebo. Patients without MRD prior to transplant did not derive the same benefit.

FLT-3 inhibitors have proven to be very effective in the treatment of patients with FLT-3 mutated AML both in the upfront and relapsed setting. Further trials are needed to fully understand exactly where and how to incorporate these drugs particularly in patients not fit for high intensity induction and in the post-transplant setting.

## IDH1/2

Isocitrate Dehydrogenase 1 and 2 mutations are relatively common in AML (6–16% for IDH1 and 8–19% for IDH2) [37]. IDH1 and 2 mutations typically occur in the enzymatic active sites, R132 and R140 loci of IDH1 and R172 locus of IDH2 [38]. Normally, IDH1 and 2 catalyze the conversion of isocitrate to α-ketoglutarate, generating NADH in the process. Mutations in these enzymes result in decreased conversion of isocitrate to α-ketoglutarate (α-KG) and a decrease in the NADPH dependent reduction of α-KG to 2-hydroxyglutarate (2-HG) [39]. The accumulation of 2-HG in cells results in the competitive inhibition of α-KG dependent processes within the cell. Primarily, 2-HG inhibits TET2 dependent cytosine 5-hydroxymethylation of DNA, resulting in characteristic hypermethylation pattern in IDHm leukemic stem cells [40]. Additionally, 2-HG accumulation also results in inhibition of cytochrome c oxidase (COX) resulting in a lower threshold for triggering apoptosis in response to BCL-2 inhibition [41].

The prognostic impact of IDH1/2 mutations in AML is controversial, with multiple studies demonstrating conflicting impacts on prognosis [39, 42, 43]. A cohort analysis of AML patients with IDH mutations from multiple cooperative groups demonstrated that prognosis is likely impacted by patient age and co-mutations which further complicate the picture [44]. Therefore, IDH1 and 2 mutations are not currently included in ELN 2022 risk stratification for AML.

Multiple small molecule inhibitors of IDH1/2 have been studied in the upfront and R/R setting. The first IDH inhibitor to be approved for AML was Enasidenib in 2017. The approval was based on the results of a single arm Phase 1/2 study of single agent Enasidenib for IDH2m, R/R AML patients with encouraging response rates [45]. Since that time, Enasidenib has been studied in combination with Azacitidine in patients with ND IDH2m AML unfit for intensive chemotherapy. In a phase 1b/2 trial, Enasidenib + Azacitidine was compared to Azacitidine alone [46]. CR and ORR (53% vs 12% and 71% vs 42% respectively) were significantly longer with the combination therapy, however OS was not significantly prolonged. This is possibly due to the significant number of patients in single arm Azacitidine who were treated with Enasidenib at progression. The triplet combination Azacitidine/Venetoclax/Enasidenib was also evaluated in a small group of patients with safety comparable to Enasidenib/Azacitidine alone, with CRc 86% and 1-year OS rate of 67% in the relapsed refractory setting, which compares favorably to response rates to Enasidenib/Azacitidine alone [47].

There are two approved IDH1 inhibitors, Ivosidenib and Olutasidenib. Unlike Enasidenib and Olutasidenib, which are only approved in the R/R setting, Ivosidenib is approved for both the ND (unfit for intensive chemotherapy) and R/R setting in patients with IDH1m AML. Ivosidenib was approved both as monotherapy and in combination with Azacitidine for ND AML. Ivosidenib’s approval for R/R IDHm AML was based on encouraging single agent response rates from a phase 1 dose escalation/expansion trial [48]. Since that time, Ivosidenib has also been studied in combination with Azacitidine in the AGILE trial. In this phase III trial, 146 patients with ND IDH1m AML, unfit for intensive chemotherapy, were randomized to Ivosidenib + Azacitidine vs Azacitidine alone. Patients had higher CR rates (47% vs 15%) and significantly longer survival with the combination (24 vs 7.9 months, *p* = 0.001) [49]. Finally, the results of a study evaluating the safety and efficacy of the triplet combination Ivosidenib/Azacitidine/Venetoclax were recently published [50]. 67 patients with either MDS/MPN, and R/R and ND AML were enrolled. CRc rates were encouraging with 93% and 63% response in patients with ND and R/R AML respectively. OS was also encouraging with 67% ND AML patients alive at 24 months and a median OS of 9 months in R/R patients.

Olutasidenib was approved in the R/R setting based on the results of a single arm study of 153 patients with CR rate 32% and ORR 48% with excellent median duration of CR/CRh of 25.9 months, which compares favorably to the DOR of 8.2 months, seen with single agent Ivosidenib [51]. OS was 11.6 months in the overall population, again comparing favorably to single agent Ivosidenib, with the caveat that there is no randomized data comparing the two drugs directly with each other. Olutasidenib has also been studied in combination with Azacitidine in the R/R setting with median OS of the combination 12.1 months [52].

Ivosidenib and Enasidenib have been studied in early phase trials in combination with intensive chemotherapy for patients with newly diagnosed de novo and secondary AML. A phase 1 trial reported favorable safety profiles with CR/CRi rates of 77% and 74% for Ivosidenib and Enasidenib respectively [53]. Further trials of these drugs in combination with intensive chemotherapy are ongoing.

IDH inhibitors are typically well tolerated but do carry unique toxicities. Both IDH1 and 2 inhibitors carry risks of differentiation syndrome typically characterized by dyspnea, culture-negative fevers, pulmonary infiltrates, and/or hypoxia. This is usually manageable with treatment interruptions and steroids. Enasidenib carries a toxicity of hyperbilirubinemia due to off-target UGT1A1 inhibition. Ivosidenib, but not Olutazidenib, carries a risk of QTc prolongation which may require treatment interruption/dose reduction [54].

Further research is needed to characterize the optimal sequencing of IDH inhibition particularly in the era of Venetoclax given that BCL-2 inhibition is uniquely effective in IDH-mutant AML.

## Menin

KMT2A (MLL1), located on chromosome 11q23, is a large DNA binding protein expressed in hematopoietic cells that is very important for normal cellular development. KMT2A binding to DNA is influenced by interactions with other proteins such as Menin, a scaffolding protein which interacts with transcription factors and other proteins which regulate gene expression [55]. The KMT2A complex regulates gene expression through histone methylation [56]. KMT2A fusions are relatively common chromosomal rearrangements seen in AML, occurring in 70–80% in infant leukemia and 5–10% in leukemias overall. It is also commonly seen in patients who develop AML after being treated with topoisomerase II inhibitors. There have been over 80 different fusion partners identified, some of which are more common in AML compared with ALL, the most common of which is t(9;11) (KMT2A-MLLT3) [57]. These fusion proteins bind to DNA/chromatin and cause leukemic transformation of hematopoietic stem cells through deregulation of genes critical for hematopoietic cell development. The most well studied of these genes are the *HOX9* gene and its cofactor *MEIS1*. Altered expression of these genes results in altered expression of various genes known to be important in leukemogenesis, and leukemic transformation [58].

The prognosis of KMT2A rearranged leukemias appears to be worse than that of AML patients with normal cytogenetics and may be influenced by the specific fusion partner [59]. ELN22 groups patients with t(9;11) in the intermediate risk category and patients with other KMT2A fusions in the adverse risk group.

Nucleophosmin (NPM1) is a nuclear chaperone protein involved in numerous cellular functions including ribosomal synthesis, stress response, and genomic stability [60]. NPM1 is one of the most commonly mutated genes in adult AML, seen in 20–30% of cases [61]. These mutations are either 4 base pair frameshift insertions or duplications of exon 12, which result in altered nuclear trafficking of NPM1 and significantly more cytosolic NPM1 protein (NPM1c) [62]. Mutations in NPM1 typically confer favorable prognosis in the absence of other alterations. It was discovered that NPM1 mutated AML is also driven by *HOX* gene overexpression similar to that of KMT2A rearranged AML, and that growth of NPM1 mutated leukemic cells could be arrested by treating with Menin inhibitors [63, 64].

Finally, nucleoporin 98 (NUP98), located chromosome 11p15, is an essential component of nuclear porin complexes, which are normally responsible for shuttling proteins in or out of the nucleus. In addition to this function as a nuclear membrane transport protein, NUP98 has also been localized to the nucleoplasm where it may also function as a transcription factor [65]. In approximately 1–2% of adult patients with AML, fusions of NUP98 to one of more than 30 different partners, have been identified as drivers of leukemogenesis [66]. NUP98 fusions typically portend poor prognosis and may be associated with chemotherapy resistance [67, 68]. Like MLL fusion and NPM1 mutations, NUP98 fusion proteins bind to chromatin near *HOX* genes resulting in their overexpression through a number of different mechanisms, including altered DNA methylation and acetylation [69]. The binding of NUP98 fusion genes to chromatin is dependent on both MLL and Menin, and in preclinical studies, leukemic cells harboring NUP98 fusions were shown to respond to Menin inhibition [70].

There are several promising Menin inhibitors currently in clinical trials. These inhibitors prevent the interactions between the Menin/KMT2A complex with DNA/chromatin, enabling cessation of the aberrant gene expression profile and preventing leukemic transformation. The Menin inhibitor Revumenib was studied in the phase 1 AUGMENT-101 trial in patients with KMT2A rearranged and NPM1m R/R AML patients as well as KMT2Ar ALL [71•]. CR/CRh was 33% in KMT2Ar and 21% in NPM1m AML with ORR 59/36% respectively. Major toxicities included QTc prolongation and differentiation syndrome. The KOMET-001 trial was recently presented, evaluating another Menin inhibitor Ziftomenib in a similar patient population [72•]. In this trial, NPM1m patients had higher rates of CR/ORR 30%/40% than those with KMT2Ar 16.7%/33.3%. The Phase 2 trial results are eagerly awaited. A phase 1 trial evaluating a third oral Menin inhibitor, JNJ-75276617, was recently presented [73]. Fifty-six patients with R/R AML were included with ORR and CR rates similar to the other 2 inhibitors. The results of a phase 1 study evaluating the all oral combination of Revumenib, Decitabine/Cedazuridine, and Venetoclax (SAVE), were recently presented at ASH 2023 [74]. Eight patients > 12 years old, with KMT2Ar, NPM1m, or NUP98r, R/R AML were enrolled. Encouragingly, ORR was 100% with slightly over 50% CR/CRi rate and 3 out of 7 patients achieving MRD negativity. In addition, trials are now enrolling to study these drugs in combination with additional low dose and high dose chemotherapy regimens (NCT05735184, NCT05886049).

Despite these encouraging responses to Menin inhibitors, patients who respond typically relapse rather quickly if not taken to transplant. Relapse has been shown to be commonly associated with mutations in the Menin protein, which drastically reduce binding of the drugs to the Menin-KMT2A complex while preserving its DNA binding capacity [75]. These data highlight the need for 2nd generation inhibitors that can bind to Menin despite these mutations.

We expect that upon approval these drugs will soon be regularly incorporated into the treatment paradigm of patients with KMT2Ar and NPM1m AML.

## CD123

CD123 is the alpha chain of the human interleukin-3 receptor (IL-3R), which is a member of the beta common cytokine family. This cytokine family also includes GM-CSFR and others. CD123 is only minimally expressed on normal early HSCs and expression is increased as cells differentiate down myeloid or monocytic pathways [76]. On the other hand, CD123 has been shown to be more highly expressed on myeloid leukemic stem cells (LSCs) compared to normal HSCs [77]. Overexpression of CD123 on AML blasts has also been previously shown to correlate with higher blast count at diagnosis and decreased response to treatment [78]. These findings make CD123 an intriguing target for the treatment of AML.

Several therapeutic modalities targeting CD123 have been developed to try to take advantage of its expression on LSCs. First, a naked antibody target CD123 (Talacotuzumab) was developed to induce antibody mediated cellular cytotoxicity (ADCC) of leukemic stem cells with limited efficacy and increased toxicity, resulting in the termination of its development [79].

Next, an antibody drug conjugate (ADC), Pivekimab (PVEK), was developed to deliver a cytotoxic payload to CD123 expressing LSCs. Pivekimab was studied as monotherapy in R/R AML patients with favorable safety profiles, though with relatively low ORR of 20% [80]. Given the low single agent activity, PVEK has also been studied in the R/R setting in combination with Azacitidine/Venetoclax. Data for this triplet were presented in 71 patients with R/R AML with ORR 51% and CRc 31% with even higher rates in Venetoclax naive patients (62% and 47% respectively) [81]. More recently, this triplet was presented in a population of ND CD123 + AML patients. CR/CRc rates of 52%/66% were reported, and of the patients achieving CR, 73% achieved MRD negativity [82]. Safety outcomes were similar to Azacitidine/Venetoclax alone. Given encouraging response rates, survival data is eagerly awaited for this combination. In addition, a trial combining PVEK with FLAG-IDA in ND AML is currently enrolling (NCT06034470).

Another modality targeting CD123 on LSCs is Bispecific T cell engagers (BiTEs) and Natural Killer Cell Engagers (NKCEs). These are antibodies that carry domains specific to CD123, as well as to immune cell epitopes like CD3 (T cells) and CD16, NKp46 (NK cells) to mediate immune cell killing of LSCs. There have been several attempts to develop BiTEs targeting CD123, though those furthest along in development, namely Vibecomatab and Flotetuzumab, were discontinued. Several other BiTEs are in preclinical development [83]. Results of a CD123 targeting NKCE, SAR443579, for R/R AML were presented at ASCO 2023, with low rates of toxicity but also low rates of CRc (13%) with further enrollment ongoing.

CD123 targeting CAR-T cells have also been developed. Phase 1 data using an allogenic, off the shelf, CD123 CAR-T (UCART123V1.2) has been published in the Ameli-01 study. 16 patients with R/R CD123 + AML received CAR-T therapy after lymphodepletion with either Flucytosine/Cyclophosphamide (FC) or Flucytosine/Cyclophosphamide/Alemtuzumab (FCA). CAR-T expansion was more robust in the FCA arm, with one patient in the FCA arm achieving MRD negative CR and one achieving stable disease with > 90% blast reduction [84]. Several other CD123 CAR-T cells have been developed and early phase trials are underway (NCT04318678, NCT03631576). It is also worth mentioning that CAR-T cells have been developed for other targets in AML including CLL-1 and CD33, among others, with mixed results [85].

Finally, Tagraxofusp, a drug containing IL-3 ligand conjugated to the first 388 amino acids of diphtheria toxin (DT), has been studied in AML. IL-3 ligand binding to IL-3 on the cell surface results in internalization of the compound and release of DT within the cell. This results in cell death through disruption of protein synthesis by DT binding to Elongation Factor-2. As a single agent, Tagraxofusp has minimal activity in AML [86]. Given the poor response rates to single agent therapy, Tagraxofusp has been studied in combination with Azacitidine/Venetoclax in both ND and R/R AML in early phase trials. In a phase 1b study, 26 patients with ND and 11 with R/R CD123 + AML received triplet Tagraxofusp/Azacitidine/Venetoclax [87]. All ND patients were categorized as adverse risk per ELN22. ND patients achieved CR/CRc rates 39%/69% with 71% of responders achieving MRD negativity. Of all ND patients with TP53m 54% achieved CR/CRc. One response of MLFS seen in R/R setting. Given these encouraging response rates, particularly with TP53m AML, further data on this triplet is eagerly awaited.

While not yet regularly incorporated into AML treatment paradigms, CD123 targeting therapies are emerging as potential options and may be incorporated into earlier lines of treatment in the future.

## CD47

CD47 is a cell surface protein expressed on all cells in the body and is highly expressed on AML cells. This protein binds to SIRPα on macrophages resulting in a “don’t-eat-me” signal, which leukemic cells use as a way to evade the immune system. CD47 has been shown to be upregulated in LSCs and anti-CD47 antibodies have been shown to induce phagocytosis of AML stem cells by macrophages both in vitro and in vivo [88, 89].

Magrolimab is an anti-CD47 antibody that blocks this signal and results in increased phagocytosis of AML cells in vitro. The Magrolimab/Azaciditine/Venetoclax showed promising activity in a phase 1b/2 trial in both the ND and R/R settings [90]. In the ND population ORR was 80% and most strikingly ORR was 74% and CR was 41% in patients with TP53m disease, which compared favorably to historical controls. One major toxicity seen with the addition of magrolimab was anemia due to targeting of CD47 on older circulating RBCs. This anemia typically resolves quickly as new RBCs enter circulation [91].

There was initially significant excitement for Magrolimab, especially given the encouraging response rates in TP53m disease. However, the ENHANCE trial evaluating the doublet Magrolimab/Azacitidine in MDS failed to reach its primary endpoint with subsequent FDA hold on the phase 3 trial in AML. Other CD47 or SIRP1α targeting antibodies sparing red cells continue development, with Maplirpacept being furthest along.

## Conclusion

Patients with AML now have many more options in both the ND and R/R setting. While these treatments are very exciting, they only work for particular subsets of AML, and many patients have AML not targetable by most of the agents discussed in this article. In particular, patients with TP53m AML still remain a unique and difficult challenge. Hopefully, additional therapeutic targets will emerge in the near future to allow for all patients to receive tailored therapy for their AML.

## Data Availability

No datasets were generated or analysed during the current study.
